# Delayed contrast-enhanced MRI of the coronary artery wall in patients with Takayasu's arteritis: initial experience and comparison to patients with stable coronary artery disease

**DOI:** 10.1186/1532-429X-14-S1-P137

**Published:** 2012-02-01

**Authors:** Christopher Schneeweis, Bernhard Schnackenburg, Alexander Berger, Udo Schneider, Matthias Stuber, Jing Yu, Eckart Fleck, Rolf Gebker, Sebastian Kelle

**Affiliations:** 1 Internal Medicine/Cardiology, German Heart Institute Berlin, Berlin, Germany; 2 Philips Health Care, Hamburg, Germany; 3 Rheumatology, Charite, Berlin, Germany; 4 Radiology, Johns Hopkins University, Baltimore, MD, USA; 5 Radiology, Center for Biomedical Imaging and University of Lausanne, Lausanne, Switzerland

## Summary

We sought to assess late gadolinium enhancement (LGE) of the coronary artery wall and ascending aorta in patients with Takayasu arteritis (TA) compared to patients with stable CAD. Our findings suggest that LGE of the coronary artery wall can be demonstrated in patients with TA and seems to be similarly pronounced as in CAD patients. The observed coronary LGE seems rather unspecific and differentiation between coronary vessel wall fibrosis and inflammation still remains unclear.

## Background

Takayasu arteritis (TA) is a rare chronic inflammatory granulomatous arteritis of the aorta and its major branches. Late gadolinium enhancement (LGE) with magnetic resonance imaging (MRI) has demonstrated its value for the detection of vessel wall alterations of large arteries in TA [1]). Involvement of coronary arteries may appear in 10 to 30% in TA [2,3]. Recently, LGE of the coronary artery wall has been demonstrated in patients with coronary artery disease (CAD) [4]. The aim of this study was to assess LGE of the coronary artery wall and ascending aorta in patients with TA compared to patients with stable CAD.

## Methods

We enrolled 9 patients (8 female, average age 46±13 years) fulfilling the ACR criteria [5] of TA without known CAD. In the CAD group 9 patients participated (8 male, average age 65±10). CAD was defined by x-ray angiography as the presence of stenosis ≥ 50%. Studies were performed on a commercial 3T whole-body MR imaging system (Achieva; Philips, Best, The Netherlands). A 3D inversion prepared navigator gated spoiled gradient-echo sequence (TR/TE = 4.3/1.6 ms, flip angle = 20° voxel size: 1.0x1.0x2.0 mm3 reconstructed to 0.7x0.7x1mm3) was repeated 34-45 minutes after low-dose gadolinium administration.

## Results

No coronary vessel wall enhancement was observed prior to contrast in either group. Post contrast, LGE on IR scans was detected in 28 of 50 segments (56%) seen on T2Prep in TA and in 25 of 57 segments (44%) in CAD patients. Quantitative LGE analysis of the ascending aorta showed a significant increase of contrast-to-noise-ratio (CNR) between pre and post contrast agent administration in TA (5.3±4.5 vs. 13.5±5.7) and in CAD patients (2.5±2.3 vs. 4.7±2.4), with p=0.028 for both groups. A highly significant difference for CNR of the aortic wall post contrast between TA and CAD patients was observed (p=0.001). LGE quantitative assessment of coronary artery vessel wall CNR post contrast revealed no significant difference between the two groups (CNR in TA: 6.0±2.4, respectively 7.3±2.5 in CAD; p=0.474).

## Conclusions

Our findings suggest that LGE of the coronary artery wall can be demonstrated in patients with TA and seems to be similarly pronounced as in CAD patients. The observed coronary LGE seems rather unspecific and differentiation between coronary vessel wall fibrosis and inflammation still remains unclear.

## Funding

none.

**Figure 1:**
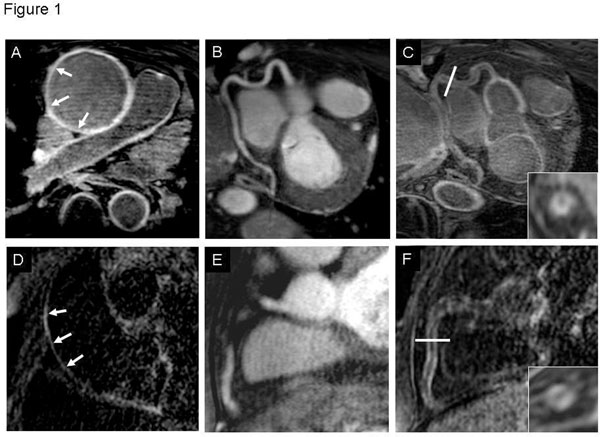
A shows an IR scan of the ascending aorta with LGE (white arrows) in the transverse plane in a TA patient. B demonstrates T2Prep scan of the RCA, while C shows the coronary IR scan in the same patient post contrast with an enlarged cross sectional view; orientation is marked in C with white bar. D shows an IR scan of the ascending aorta in the sagittal plane post contrast in a patient with CAD. In E, RCA of the same CAD patient in T2Prep and in F as IR scan with an enlarged cross sectional view is demonstrated.
